# Blockade of bovine PD-1 increases T cell function and inhibits bovine leukemia virus expression in B cells in vitro

**DOI:** 10.1186/1297-9716-44-59

**Published:** 2013-07-22

**Authors:** Ryoyo Ikebuchi, Satoru Konnai, Tomohiro Okagawa, Kazumasa Yokoyama, Chie Nakajima, Yasuhiko Suzuki, Shiro Murata, Kazuhiko Ohashi

**Affiliations:** 1 Department of Disease Control, Graduate School of Veterinary Medicine, Hokkaido University, Sapporo 060-0818, Japan; 2 Research Center for Zoonosis Control, Hokkaido University, Sapporo 001-0020, Japan

## Abstract

Programmed death-1 (PD-1) is a known immunoinhibitory receptor that contributes to immune evasion of various tumor cells and pathogens causing chronic infection, such as bovine leukemia virus (BLV) infection. First, in this study, to establish a method for the expression and functional analysis of bovine PD-1, hybridomas producing monoclonal antibodies (mAb) specific for bovine PD-1 were established. Treatment with these anti-PD-1 mAb enhanced interferon-gamma (IFN-γ) production of bovine peripheral blood mononuclear cells (PBMC). Next, to examine whether PD-1 blockade by anti-PD-1 mAb could upregulate the immune reaction during chronic infection, the expression and functional analysis of PD-1 in PBMC isolated from BLV-infected cattle with or without lymphoma were performed using anti-PD-1 mAb. The frequencies of both PD-1^+^ CD4^+^ T cells in blood and lymph node and PD-1^+^ CD8^+^ T cells in lymph node were higher in BLV-infected cattle with lymphoma than those without lymphoma or control uninfected cattle. PD-1 blockade enhanced IFN-γ production and proliferation and reduced BLV-gp51 expression and B-cell activation in PBMC from BLV-infected cattle in response to BLV-gp51 peptide mixture. These data show that anti-bovine PD-1 mAb could provide a new therapy to control BLV infection via upregulation of immune response.

## Introduction

Immunoinhibition is considered one of the reasons responsible for the refractory nature of several types of tumors and chronic infections [1,2]. One of them, bovine leukemia virus (BLV) is known to induce immunosuppression and B cell lymphoma in cattle, and lead to enormous damages to livestock industries around the world [3]. BLV establishes a chronic infection in B cells for several years until infected cattle develop B-cell lymphoma mainly in lymphoid tissue, although neither viral RNA nor protein expression was readily detected in vivo or freshly isolated lymphocytes [4,5]. During the chronic infection, the suppression of both CD4^+^ T cell proliferation and cytotoxic immune response against BLV antigens is correlated to disease progression [3,6]. To develop strategies to effectively control BLV infection, the mechanism responsible for this immunosuppression needs to be clarified.

Programmed death-1 (PD-1) has been recognized as being at the heart of peripheral immune tolerance and pathogen-specific immunoinhibition [2]. In various types of chronic infections and tumors, PD-1 and its ligand, PD-ligand-1 (PD-L1) play an important role in inhibiting chronically activated T cells specific for pathogens, resulting in the induction of “exhausted” T cells [5,7-9]. Treatment with monoclonal antibodies (mAb) specific for either PD-1 or PD-L1 reactivates exhausted immune responses such as proliferation, cytokine production, and cytotoxic capabilities of exhausted T cells ex vivo [7,10], and in vivo [11,12], and was tested in clinical trials with cancer patients [13,14].

In the field of veterinary medicine, the PD-1/PD-L1 pathway was also investigated in the pig [15,16], chicken [17] and cat [18] and found to contribute to pathogenesis and immune evasion of chronic infectious diseases. Our previous reports also showed that the expression of PD-L1 in B cells which were target cells for BLV, was upregulated in BLV-infected (BLV^+^) cattle as the disease progressed, and PD-L1 blockade upregulated expressions of *interferon-gamma* (*IFN-γ*) and *interleukin* (*IL*)*-2* mRNA in peripheral blood mononuclear cells (PBMC) in vitro [19]. The expression levels of *PD-1* mRNA were upregulated in CD4^+^ and CD8^+^ T cells isolated from BLV^+^ cattle with B-cell lymphoma (BCBL) [20].

In previous reports, anti- “human” PD-1 or PD-L1 “polyclonal” antibodies (pAb) were used to analyze their expression and to block the PD-1/PD-L1 pathway [18,19]. Under some experimental conditions, anti-PD-1 pAb induced IL-10 production by monocytes, resulting in the inhibition of CD4^+^ T cell function [21]. However, at the present time, mAb specific for animal PD-1 and PD-L1 which can reactivate exhausted immune reaction are not available, although they are essential for further investigation and development of new therapy for refractory diseases, such as BLV infection. In this study anti-bovine PD-1 mAb were established and their functional capabilities were assessed using PBMC from BLV^+^ and BLV-uninfected (BLV^-^) cattle in vitro. The upregulation of PD-1 expression was found in CD4^+^ and CD8^+^ T cells isolated from BCBL. The treatment with an anti-PD-1 mAb upregulated IFN-γ production and reduced both B cell activation and BLV-gp51 expression in PBMC isolated from BLV^+^ cattle. These data suggest that anti-PD-1 mAb can be applicable for antibody drug to control BLV infection.

## Materials and methods

### Construction and expression of recombinant soluble bovine PD-1-immunoglobulin fusion protein

Soluble PD-1-bovine IgG1 fusion protein (PD-1-Ig) was expressed in a mammalian cell expression system. The extracellular domain fragment of bovine PD-1 was amplified and the fragment was inserted into the cloning site of a modified pCAGGS (provided by Dr J. Miyazaki, Osaka University; [22]) that contained a mouse CD150 leader sequence at the N terminus and the Fc fragment of bovine IgG1 at the C terminus [23]. PD-1-Ig was produced in Cos-7 cells transfected transiently by Lipofectamine 2000 (Life Technologies, Carlsbad, CA, USA) and purified from the media with Protein G Sepharose 4 Fast Flow (GE Healthcare UK Ltd, Buckinghamshire, UK) according to the manufacturer’s protocol. The expression and purification of PD-1-Ig was confirmed by sodium dodecyl sulfate-polyacrylamide gel electrophoresis (SDS-PAGE) and enzyme-linked immunosorbent assay (ELISA) using anti-bovine IgG-Fc (Rockland Immunochemicals, PA, USA).

### Generation and screening of mAb specific for bovine PD-1

To obtain mAb specific for bovine PD-1, a rat was immunized with about 66 μg of PD-1-Ig and complete Freund adjuvant. Nineteen days later, 100 μg of PD-1-Ig was shot into the rat as a boost. On the 24^th^ day of the first immunization, lymphocytes isolated from the iliac lymph node (LN) were fused with myeloma cells and cloned, and the supernatants from hybridomas were screened by ELISA for the reactivity of culture supernatant with PD-1-Ig. Clones that produce antibodies specific for bovine IgG1-Fc were excluded by ELISA using bovine IgG as an antigen. The immunization of rats and ELISA was performed at Cell Engineering Corporation (Osaka, Japan). The hybridomas were also screened by flow cytometry using Cos-7 cells that were transfected with bovine PD-1 coding pCMV-Tag-1 (Agilent Technologies, CA, USA). Hybridomas producing antibodies that reacted with transfected but not untransfected PD-1 Cos-7 cells were cloned by limiting dilution.

### Western blotting

To test the reactivity of anti-bovine PD-1 mAb, Western blot analysis was performed using Chinese hamster ovary (CHO)-DG44 cells (provided by Dr Y. Suzuki) that stably express bovine PD-1. CHO-DG44 cells were transfected with bovine PD-1 coding pCMV-Tag-1 using Lipofectamine Ltx (Life Technologies). Transfectants were selected in CD-DG44 (Life Technologies) containing G418 sulfate (Wako, Osaka, Japan; 800 μg/mL) for two weeks. The cells were then incubated with anti-bovine PD-1 mAb (produced from our hybridoma, 5D2), followed by incubation with Anti-Rat IgG Micro beads (Miltenyi Biotec, Bergisch Gladbach, Germany). CHO-DG44 cells highly expressing PD-1 were sorted by auto MACS Pro (Miltenyi Biotec), cultivated for a week, and then re-sorted. CHO cells transfected with non-coding pCMV-Tag-1 were selected in the same way and used as a negative control. The cells were lysed in 2× SDS buffer (125 mM Tris–HCl pH 6.8, 4% SDS, 10% 2-mercaptoethanol, and 20% glycerol) and boiled for 10 min. The samples were separated on 12% SDS-polyacrylamide gels and transferred onto a polyvinylidene difluoride membrane (Merck Millipore, MA, USA). After being blocked with 3% skim milk in phosphate-buffered saline (PBS, pH 7.2) containing 0.05% Tween 20 (PBS-T), the membranes were incubated at room temperature for 2 h with anti-PD-1 mAb (2C12 and 3G2: 3 μg/mL, 2H7 and 5D2: 1 μg/mL), followed by washing and incubation with horse radish peroxidase (HRP)-conjugated anti-rat IgG (MP Biomedicals, CA, USA). The membrane was also probed with anti-Actin antibody (Merck Millipore; clone C4) and anti-myc tag antibody (Abcam, Cambridge, UK; goat polyclonal antibody) as loading control and positive control. After washing, the membranes were incubated with Immobilon Western Chemiluminescent HRP Substrate (Merck Millipore) to visualize signals, and analyzed with a Fluor-S Multi Imager (Bio-Rad Laboratories, CA, USA).

### Samples from cattle and BLV diagnosis

Blood and mesenteric LN samples from Japanese black and Holstein-Friesian were investigated. In this study, we obtained blood samples from 95 cattle altogether (BLV+: 53, BLV-: 42) bred on several farms. They were collected in about ten installments. Peripheral venous blood was collected from cows into tubes containing sodium heparin (Ajinomoto, Tokyo, Japan). All of the cattle from which the blood samples were obtained had been diagnosed with BLV infection by nested PCR as described previously [19] at the Veterinary Teaching Hospital, Graduate School of Veterinary Medicine, Hokkaido University between 2008 and 2012. LN samples were provided by the Meat inspection center and veterinary hospitals in Japan, and diagnosed by nested-real time PCR using primers described previously [19]. The first amplification was conducted by KOD FX neo (Toyobo, Osaka, Japan) using less than 20 000 LN cells as templates, and the second amplification was performed using SYBR Premix DimerEraser (Takara, Shiga, Japan). This study was conducted in accordance with the guidelines of the Institutional Animal Care and Use Committee of Hokkaido University, Japan. Samples were collected after informed consent was obtained from farmers.

To diagnose B cell lymphoma, phenotypic analysis of PBMC and/or tumor-bearing LN (mesenteric, iliac, gastric or superficial cervical LN) cells from BCBL which had been diagnosed clinically were performed by flow cytometry. Double staining were conducted using anti-bovine IgM (BIG73A; VMRD, WA, USA) pre-labeled with Zenon Alexa Fluor 488 (Life Technologies) and the following antibodies; anti-WC4 (CC55; CD19 like; AbD Serotec, Oxford, UK) [24], anti-B-B7 (GB25A; CD21 like; VMRD) and anti-bovine CD5 (CACT105A; VMRD). Alexa Fluor 647-conjugated anti-mouse IgG (Life Technologies) was used for the antibodies other than anti-IgM as a secondary antibody. The samples in which more than 85% of the cells were B cells (IgM^+^ CD5^+^, IgM^-^ WC4^+^ or IgM^-^ CD21^+^) were diagnosed as B-cell lymphoma induced by BLV. More than 30 000 lymphocytes were analyzed.

### Cell preparation from blood and lymph nodes

PBMC were purified from the blood samples by density gradient centrifugation on Percoll (GE Healthcare UK Ltd). LN tissues were minced in PBS and passed through a cell strainer (40 μm; BD Biosciences, NJ, USA).

### PD-1 expression analysis

**Figure 1:**
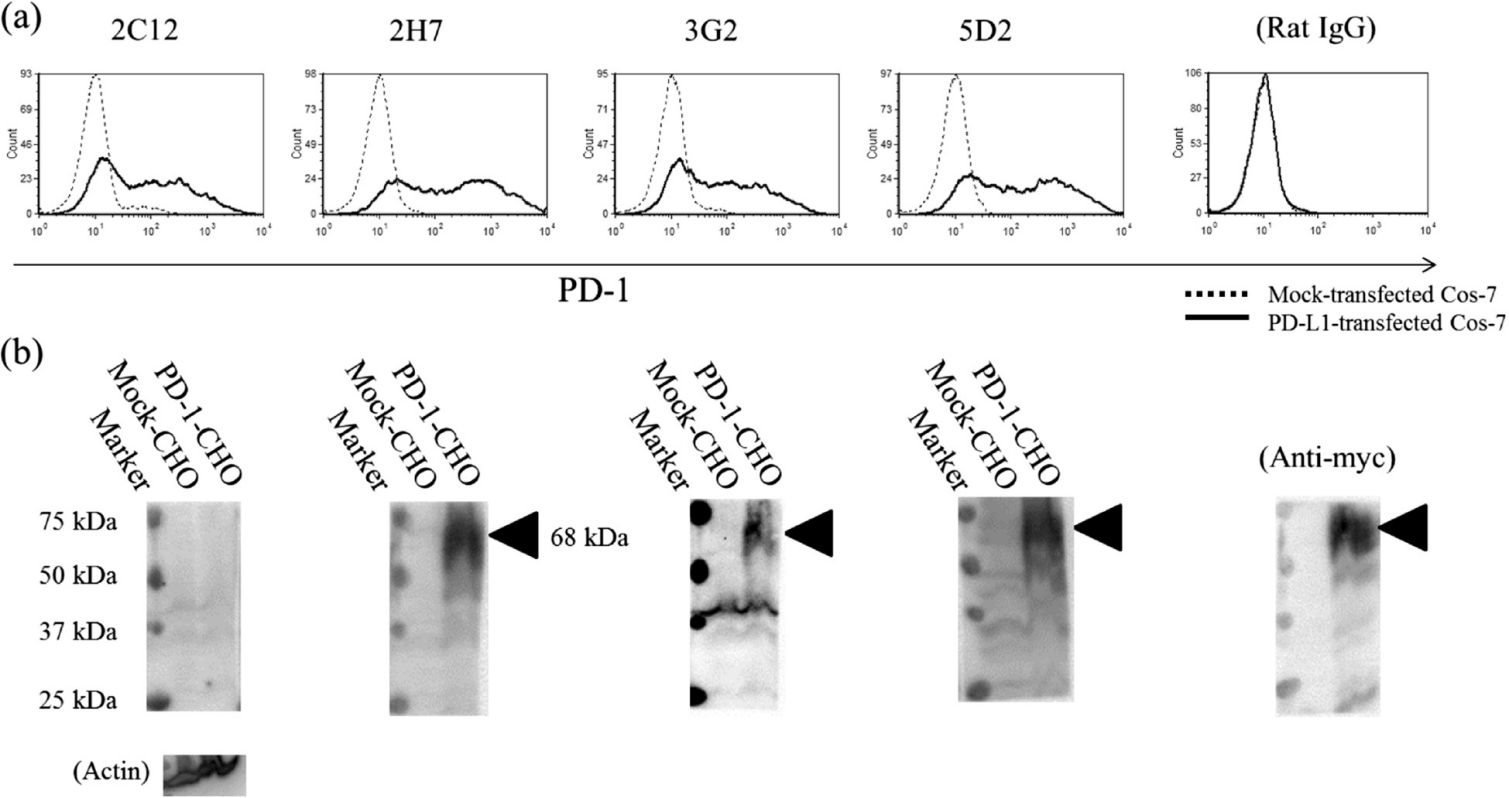
**Recognition of PD-1-expressing cells by anti-PD-1 mAb. (a)** Flow cytometric analysis of surface expression of bovine PD-1. Cos-7 expressing PD-1 (black line) and Cos-7 transfected with the control vector (dashed line) were stained with four types of anti-PD-1 mAb (2C12, 2H7, 3G2, and 5D2) and isotype control (Rat IgG). **(b)** Western blotting analysis of bovine PD-1 expression in CHO-DG44 cells stably expressing bovine PD-1. Three types of anti-PD-1 mAb recognized PD-1 (triangle) at about 68 kDa. Anti-actin antibody and anti-myc antibody was used as a loading control and a positive control.

**Figure 2:**
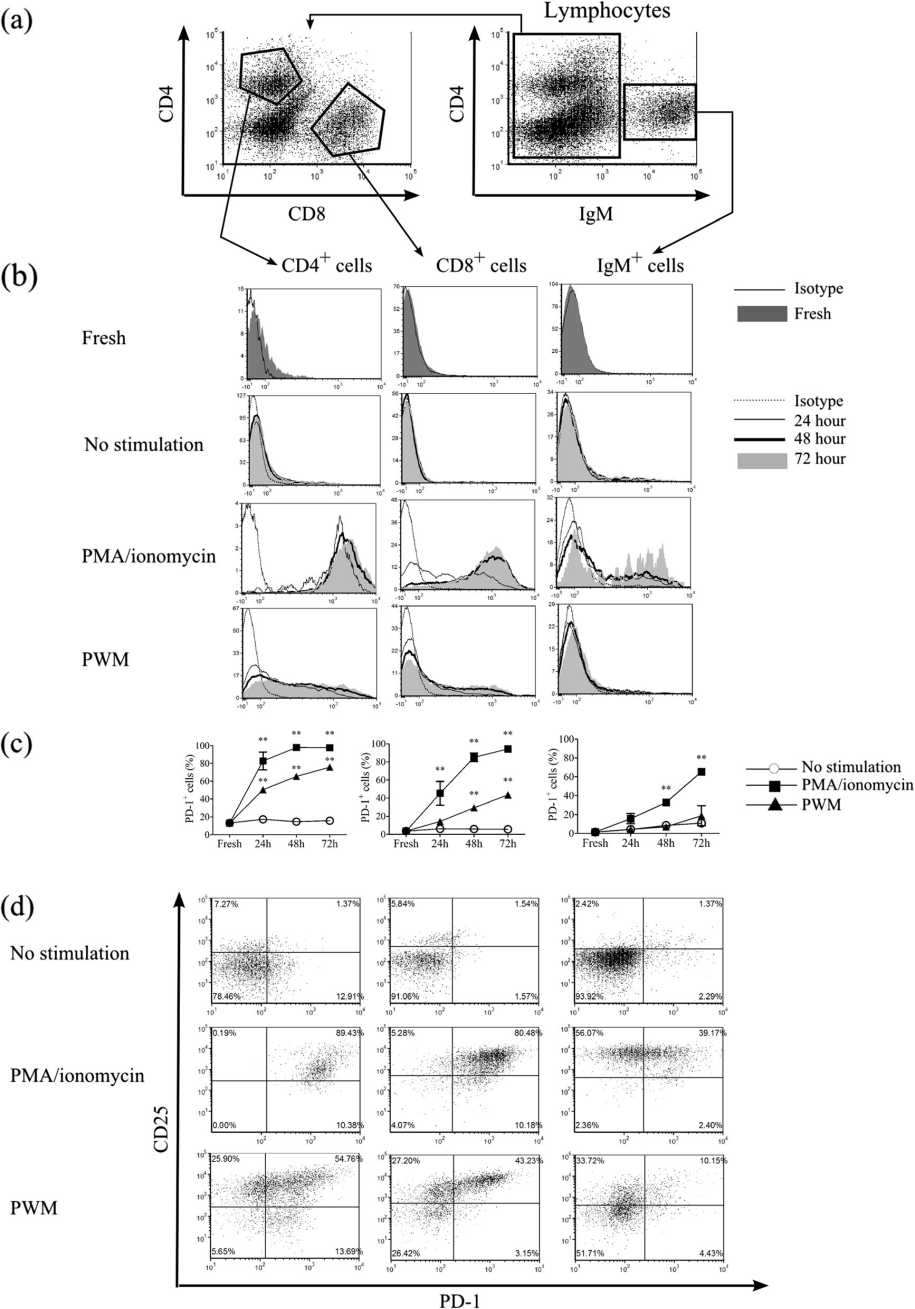
**Recognition of PD-1-expressing lymphocytes by anti-PD-1 mAb. (a)** Gating strategies for CD4^+^ T cells, CD8^+^ T cells and IgM^+^ B cells in bovine lymphocytes. **(b)** Representative histograms obtained by flow cytometry of PD-1 expression in CD4^+^ T cells, CD8^+^ T cells and IgM^+^ B cells isolated from three BLV^-^ cattle. Freshly isolated PBMC were stained with anti-PD-1 (5D2), CD4, CD8, and IgM Ab. PBMC were cultivated with PBS (No stimulation), PMA/ionomycin, and PWM for 24, 48, and 72 h, and stained in a similar way. **(c)** Proportions of PD-1 positive cells in CD4^+^ T cells, CD8^+^ T cells and IgM^+^ B cells. Statistical comparisons between percentages of PD-1 positive cells stimulated with PBS and PMA/ionomycin or PWM were made using two way ANOVA. Differences were considered statistically significant at *P* < 0.05 (** *P* < 0.01). **(d)** Representative dot plots of PD-1 and CD25 expression in CD4^+^ T cells, CD8^+^ T cells and IgM^+^ B cells 48 h after cultivation.

To block nonspecific staining, 5–20 × 10^5^ PBMC were incubated in PBS containing 10% goat serum (Sigma-Aldrich, MO, USA) at room temperature for 15 min. The cells were then washed and stained for PD-1, CD4, CD8, IgM and CD25 for 30 min at room temperature. The following antibody conjugates were used in Figure 1: PD-1:5D2; CD4:CACT138A (VMRD) pre-labeled with Zenon Alexa Fluor 488; CD8:CACT80C (VMRD) pre-labeled with Lightning-Link PerCP/Cy5.5 (Innova Biosciences, Cambridge, UK); IgM:BIG73A pre-labeled with Zenon PE (Life Technologies); CD25:CACT116A pre- labeled with Lightning-Link PE-Cy7 (Innova Biosciences). In Figure 2, the cells were stained with the following antibody: PD-1, CD4 pre-labeled with Zenon Alexa Fluor 488, CD8 pre-labeled with Lightning-Link PerCP/Cy5.5, and IgM pre-labeled with Lightning-Link PE-Cy7. After washing with PBS containing 10% goat serum, the cells were incubated with APC-conjugated anti-rat IgM + IgG (Beckman Coulter, CA, USA) for 30 min at room temperature. The cells were then washed and immediately analyzed by FACS Verse (BD Biosciences) and FCS Express 4 (De Novo Software, CA, USA). No fewer than 40 000 lymphocytes and no more than 160 000 lymphocytes were analyzed among the samples.

To measure the upregulation of PD-1 expression in stimulated bovine lymphocytes isolated from BLV^-^ cattle, PBMC were cultivated for 24, 48, and 72 h at 37 °C with 5% CO_2_ in RPMI-1640 in the presence of pokeweed mitogen (PWM, 5 μg/mL, Sigma-Aldrich) or phorbol 12-myristate acetate (PMA) and ionomycin (20 ng/mL and 1 μg/mL; Sigma-Aldrich). No fewer than 13 000 lymphocytes and no more than 36 000 lymphocytes were analyzed among the samples.

### Analysis of PD-L1 expression in BLV-gp51 positive cells

To confirm PD-L1 expression in BLV^+^ cells, intracellular staining was performed. PBMC isolated from BLV^+^ or BLV^-^ cattle were cultivated for 18 h, and the cells were stained by anti-human PD-L1 (Santa Cruz Biotechnology, CA, USA) and anti-bovine IgM as previously described [19]. After surface staining, the cells were fixed and permealized by FOXP3 Fix/Perm kit (BioLegend, Cambridge, UK) according to the manufacturer’s protocol. Then, the cells were stained with anti-gp51 (BLV1; VMRD) pre-labeled with Zenon PE, washed and immediately analyzed. More than 30 000 lymphocytes were analyzed.

### PD-1 blockade assay

To determine the effect of the immune activation by anti-PD-1 mAb, bovine PBMC were cultured with 20 μg/mL of anti-PD-1 mAb or rat IgG (Sigma-Aldrich) in the presence or absence of PMA/ionomycin or in the presence of 10 μg/mL BLV-gp51 peptide mixture (15 mer). The peptide mixture covering the entire length of gp51 and overlapping by 11 amino acids, were synthesized in Hokkaido System Science (Hokkaido, Japan). Flag peptide (DYKDDDDK) was used as the negative control for gp51 peptide mixture. After 2 days, the cells were collected for detection of apoptosis and expression analysis of gp51, WC4 and CD80. The supernatants were harvested and analyzed by ELISA. For real-time PCR or cell proliferation assay, cultivated cells were collected after 1 day or 5 days. In a portion of experiments, isolated B cells from PBMC were cultured. PBMC were incubated with anti-bovine IgM, and IgM^+^ B cells were isolated by autoMACS Pro and anti-mouse IgG1 MicroBeads (Miltenyi Biotec).

### IFN-γ and IL-10 ELISA

The production of IFN-γ in supernatants was measured by ELISA for bovine IFN-γ (Mabtech, Nacka Strand, Sweden) according to the manufacturer’s protocol. The results were calculated based on a standard curve ranging from 7.8 pg/mL to 500 pg/mL. Sandwich ELISA of IL-10 was performed with two antibodies; anti-IL-10 (CC318; AbD Serotec) as capture antibody and biotin-conjugated anti-IL-10 (CC320; AbD Serotec) as the detective antibody. Briefly, 96 well plates were coated overnight with CC318 diluted with PBS. After washing with PBS and blocking by PBS-T containing 0.1% bovine serum albumin (Sigma-Aldrich), the samples were incubated in the wells for 2 h. Following washing, diluted detective antibodies (CC320) were added to the wells and incubated for 1 h. After further washing, Neutra-Avidin-HRP was added and incubated for 1 h. Finally, the plates were washed and incubated with TMB One Component Substrate (Bethyl Laboratories, TX, USA), and absorbance was measured by MTP-650FA (Corona Electric, Ibaraki, Japan). A standard curve was constructed using plasma which was separated from a cattle-derived blood stimulated with 10 μg/mL concanavalin A (Sigma-Aldrich) and 10 μg/mL lipopolysaccharide (Sigma-Aldrich) for 48 h. The 1 (64) arb. unit is defined in terms of the amount of IL-10 in 15.625 (1000) μL of the stimulated plasma. Reported values represent the mean of duplicate samples.

### Cell proliferation assay

To investigate the effect of PD-1 blockade on the proliferation of PBMC, a CFSE proliferation assay was performed. In summary, PBMC were incubated with 2 μM CFSE (Life technologies) diluted with PBS at 37 °C for 15 min, washed with RPMI-1640 three times and cultured for 5 days. Then, the cells were stained with anti-IgM pre-labeled with Lightning-Link PE-Cy7 and analyzed immediately by flow cytometry. Percentages of CFSE^lo^ IgM^-^ lymphocytes were measured for evaluation of the proliferation of lymphocytes other than B cells. No fewer than 2500 and no more than 14 000 lymphocytes other than B cells were analyzed among the samples.

### Expression analysis of BLV-gp51, WC4 and CD80

To analyze the effect of PD-1 blockade on B cell function, cultivated PBMC were stained with anti-IgM pre-labeled with Zenon Alexa Fluor 488 and anti-WC4 or anti-CD80 (AbD Serotec) or anti-BLV-gp51. Alexa Fluor 647-conjugated anti-mouse IgG was used for anti-WC4 and anti-CD80 as a secondary antibody. BLV-gp51 staining of either PBMC or isolated B cells was performed as described above. Appropriate isotype controls were performed with each sample. More than 20 000 lymphocytes were analyzed.

### Real-time PCR

Total RNA was extracted from cultivated PBMC by RNeasy Plus Mini Kit (QIAGEN, CA, USA), and cDNA was synthesized by Reverse Transcriptase M-MLV (Takara) following the manufacturer’s instructions. Quantitative RT real-time PCR was performed using the LightCycler 480 system II (Roche Diagnostics, Mannheim, Germany) according to the manufacturer’s instructions. Primers used for the amplification of *BAFF* cDNA were 5’- CCA AGC TGG AGG AAG GAG ATG AAC TC-3’ and 5’- CTC CAT CTC GGG ATA TCT TAG CAT C-3’. The amount of *BAFF* mRNA expression was divided by the expression of *GAPDH* and β*-actin* mRNA as internal control genes. Each amplification procedure was done in duplicate, and the results were indicated as relative change to control (no antibody treatment).

### Detection of apoptosis

To detect apoptotic B cells, cultivated PBMC were stained with anti-IgM as a first antibody and Alexa Fluor 647-conjugated anti-mouse IgG as a secondary antibody. Following washing, cells were incubated with Annexin V-FITC (Beckman Coulter) for 15 min and added 7-AAD (BD Biosciences). No fewer than 12 000 lymphocytes and no more than 20 000 lymphocytes were analyzed among samples. The results are presented as percentages of FITC^+^ 7-AAD^-^ cells in total IgM^+^ B cells.

### Statistical analysis

The Spearman rank-correlation, one-way ANOVA with Tukey’s post test, two-way ANOVA and Wilcoxon matched pairs test were performed using GraphPad Prism version 5.0. *P* values < 0.05 were considered statistically significant.

## Results

### Anti-PD-1 mAb react with PD-1-expressing cells

Supernatants containing antibody from 576 hybridoma colonies were screened for their binding to bovine IgG and PD-1-Ig by ELISA. Hybridomas that produced mAb-recognizing bovine IgG were excluded. Four hybridomas (2C12, 2H7, 3G2, and 5D2) were cloned and confirmed as clones producing mAb that reacted with Cos-7 expressing PD-1 but not with cells transfected with the control vector (Table 1 and Figure 1a). Control antibody (Rat IgG) did not react with both of the cells. The degree of fluorescent signals of 2H7 and 5D2 were different from those of 2C12 and 3G2. Anti-PD-1 mAb, except for 2C12, were able to recognize heat-denatured PD-1 at approximately 68 kDa by Western blotting, showing that 2C12 could recognize a conformational epitope of bovine PD-1 (Figure 1b).

**Table 1:** The number of positive hybridomas in each screening test

**ELISA for PD-1-Ig**	**ELISA for bovine IgG**	**ELISA for PD-1-Ig (did not recognize bovine IgG)**	**FACS for PD-1 expressing Cos-7**
47	78	14	4

### Anti-PD-1 mAb react with bovine lymphocytes

To confirm that anti-PD-1 mAb can recognize bovine PD-1 naturally expressed on bovine lymphocytes, we examined surface PD-1 expression on CD4^+^ and CD8^+^ T cells as well as IgM^+^ B cells freshly isolated or stimulated by mitogens, such as PWM and PMA/ionomycin in vitro. An example of the gating strategy is shown in Figure 2a. IgM^+^ B cells were first gated and IgM^-^ cells further analyzed for CD4 and CD8 expression. Amongst the four anti-PD-1 mAb, the strongest fluorescence was observed in PBMC stained with 5D2 (data not shown); therefore, the cells were stained with 5D2 for subsequent expression analyses of bovine PD-1. PD-1 was expressed mainly on CD4^+^ T cells from freshly isolated PBMC, whereas CD8^+^ T cells and B cells barely expressed PD-1 (Figure 2b).

When PBMC were cultivated in the presence of mitogen, PD-1 expression in lymphocytes was elevated, whereas in vitro incubation without any stimulant did not affect PD-1 expression in any population of lymphocytes (Figure 2b, c). PMA/ionomycin stimulation more quickly induced PD-1 expression than in the case of no stimulant, which was strongly enhanced in more than 90% of CD4^+^ and CD8^+^ T cells and half of B cells at 72 h (Figure 2b). Although PWM stimulates both T and B cells [25] like PMA/ionomycin, on one level or another, subsets of CD4^+^ and CD8^+^ T cells stimulated by PWM expressed less PD-1 antigen than PBMC treated with PMA/ionomycin at the same point in time, and the rate of the upregulation in PWM stimulation was also slower. Furthermore, upregulation of PD-1 expression on B cells was not induced by PWM stimulation. In the case of any stimulation, CD4^+^ T cells were prone to more quickly upregulate PD-1 expression than CD8^+^ T cells and B cells.

A previous report revealed that activated lymphocytes express PD-1 antigen in mice [26]; therefore bovine PD-1 expression on stimulated lymphocytes detected by a classical activation marker, CD25, was evaluated. Forty-eight hours after PMA/ionomycin stimulation, CD25 expression was strongly upregulated in either CD4^+^ and CD8^+^ T cells and IgM^+^ B cells, and almost all T cells and subset of B cells expressed PD-1 (Figure 2d). Otherwise, under PWM stimulation, CD25 expression varied widely among the subsets. Moreover, CD4^+^ CD25^-^ T cells expressed PD-1, but CD8^+^ CD25^-^ T cells barely did. We examined PD-1 expression in PBMC isolated from three healthy cattle and similar results were obtained in each case.

### Anti-PD-1 mAb activate IFN-γ production

**Figure 3:**
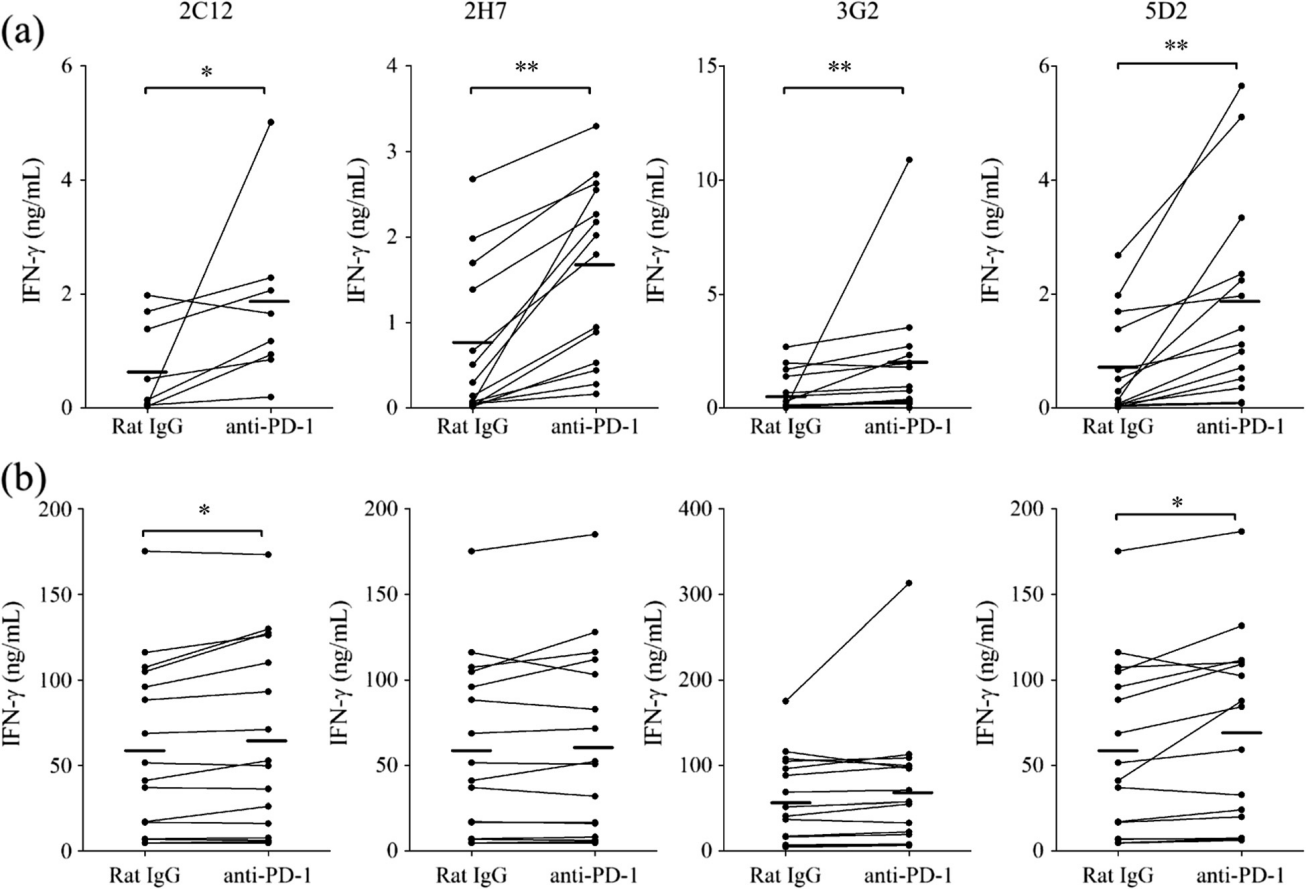
**Enhancement of cytokine production by anti-PD-1 mAb in bovine lymphocytes.** PBMC were cultivated with rat IgG control or four types of anti-PD-1 mAb (20 μg/mL) in the absence (**a**; *n* = 8 or 14) or presence (**b**; *n* = 16) of PMA/ionomycin. IFN-γ production was measured by ELISA. Statistical comparisons between rat IgG control and anti-PD-1 mAb were made using the Wilcoxon matched-pairs test. Differences were considered statistically significant at *P* < 0.05 (*P < 0.05; ** *P* < 0.01).

To clarify the function of mAb that inhibit the PD-1/PD-L1 pathway and the inhibitory signal from PD-1, we performed a PD-1 blockade assay. PBMC isolated from BLV^-^ cattle were cultured for 48 h in the presence of each anti-PD-1 mAb or control antibody, and IFN-γ production was then measured in the supernatants using ELISA. All of the four anti-PD-1 mAb significantly increased IFN-γ production in PBMC in comparison to those treated with control rat IgG (Figure 3a). The treatments of 2H7 and 5D2 upregulated the production of IFN-γ in all sample cases. Furthermore, in PBMC cultivated with PMA/ionomycin in vitro, 2C12 and 5D2, but not 2H7 and 3G2, significantly enhanced IFN-γ production (Figure 3b), showing that the function of activated lymphocytes by PMA/ionomycin stimulation could be additionally enhanced by PD-1 blockade with 2C12 or 5D2 treatment. The most effective mAb was not determined because there were individual differences in INF-γ production, and no significant difference was observed within the degree of enhancement of IFN-γ production induced by the treatment with the four mAb.

### PD-1 expression is upregulated in CD4^+^ and CD8^+^ T cells in BLV^+^ cattle with lymphoma

**Figure 4:**
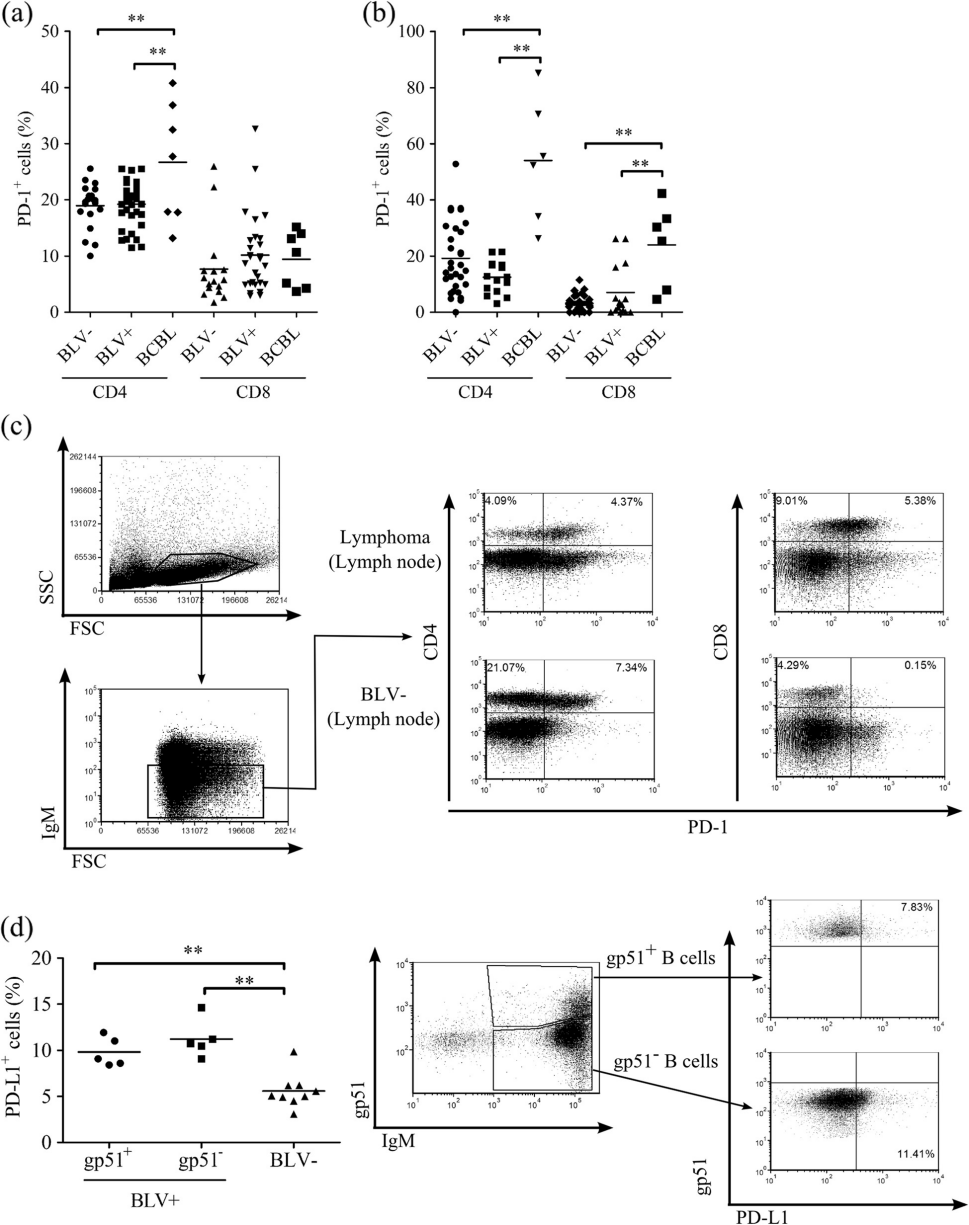
**Expression analysis of bovine PD-1 in BLV-infected cattle. (a, b)** Percentages of PD-1-expressing CD4^+^ and CD8^+^ T cells in PBMC **(a)** and LN **(b)** isolated from BLV^-^ cattle (CD4^+^ T cells; *n* = 20 and 31, CD8^+^ T cells; *n* = 16 and 31), BLV^+^ cattle (CD4^+^ T cells; *n* = 35 and 15, CD8^+^ T cells; *n* = 28 and 15) and BCBL (CD4^+^ T cells; *n* = 7 and 6, CD8^+^ T cells; *n* = 7 and 6). **(c)** An example of gating strategies for PD-1 expression in CD4^+^ T cells and CD8^+^ T cells isolated from lymph node of BCBL and BLV^-^ cattle. Values in the quadrant indicate the percentage of the cells in lymphocytes other than IgM^+^ B cells. **(d)** Percentages of PD-L1-expressing gp51^+^ (*n* = 5) and gp51^-^ (*n* = 5) in IgM^+^ B cells from BLV^+^ and BLV^-^ cattle (*n* = 9). Representative dot plots of PD-L1 staining in gp51^+^ or gp51^-^ B cells are also shown. Statistical comparisons were made using one-way ANOVA with Tukey’s test. Differences were considered statistically significant at *P* < 0.05 (** *P* < 0.01).

Our previous reports claimed that *PD-1* mRNA expression in T cells isolated from BCBL was higher than from BLV^-^ cattle [20]. To confirm the PD-1 expression on the cell surface of T cells in BLV^+^ cattle, flow cytometric analysis was performed in PBMC and mesenteric (BLV^-^ and BLV^+^ cattle) or tumor-bearing (BCBL) LN cells using anti-PD-1 mAb, 5D2. In blood, the mean percentages of PD-1^+^ CD4^+^ T cells were higher in cattle with lymphoma than in BLV^+^ and BLV^-^ cattle (Figure 4a). Meanwhile, in LN cells, the rates of PD-1 expression in both CD4^+^ and CD8^+^ T cells were significantly higher in BCBL than in BLV^+^ and BLV^-^ cattle (Figure 4b; an example of the gating strategy is shown in Figure 4c). IgM^+^ B cells in all samples barely showed PD-1 expression (data not shown), although CD19^+^ B cells in human patients of chronic lymphocytic leukemia strongly expressed PD-1 [27].

PD-L1 expression analysis in BLV infection had been performed in our previous report [19]. However, the question remained whether BLV^+^ B cells actually express PD-L1 or not. To activate the expression of the BLV protein, such as gp51 and to permit detection of the BLV^+^ B cells, PBMC were cultivated overnight before cell staining. BLV-gp51 expression was not detected in freshly isolated B cells, cultivation of PBMC overnight resulted in reactivation of gp51 expression (see Additional file 1) in line with previous findings [28-30]. The PD-L1 expression was able to be detected in gp51^+^ B cells (Figure 4d). There is no difference in percentages of PD-L1 expression between gp51^+^ and gp51^–^ B cells in BLV^+^ cattle. The mean percentages of PD-L1^+^ cells in B cells from BLV^+^ cattle were higher compared to those from BLV^-^ cattle, as described in a previous report [19].

### PD-1 blockade upregulates T-cell function in PBMC from BLV^+^ cattle

**Figure 5:**
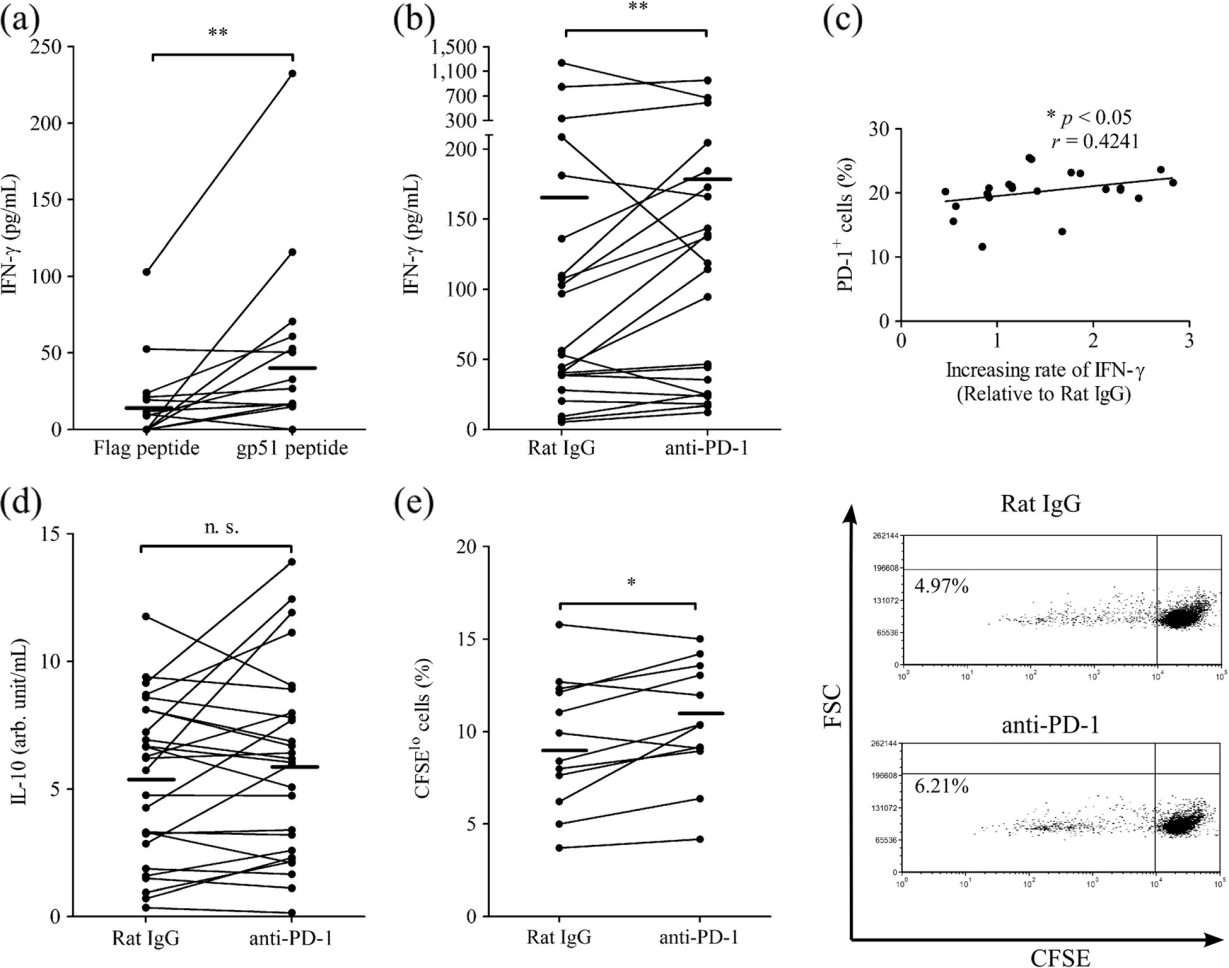
**Effect of PD-1 blockade on T-cell function. (a)** PBMC from BLV^+^ cattle were cultured with Flag peptide as negative control and gp51 peptide mix. IFN-γ production was measured by ELISA (*n* = 13). **(b, c, d)** PBMC were cultured with rat IgG control or anti-PD-1 mAb (5D2; 20 μg/mL) in the presence of gp51 peptide mix. IFN-γ and IL-10 production was measured by ELISA (**b**; *n* = 22, d; *n* = 26). Positive correlation between increasing rate of IFN-γ production and percentages of PD-1^+^ cells in CD4^+^ T cells corresponding to Figure 4a (**c**; *n* = 22). Correlation statistics were analyzed using the Spearman correlation. **(e)** The proliferative responses were evaluated by detection of CFSE^lo^ cells in IgM^-^ lymphocytes by flow cytometry (*n* = 12). Representative dot plots of CFSE-staining in lymphocytes other than B cells are shown. Statistical comparisons between rat IgG control and anti-PD-1 mAb were made using the Wilcoxon matched-pairs test. Differences were considered statistically significant at *P* < 0.05 (* *P* < 0.05; ** *P* < 0.01).

To assess whether PD-1 blockade activates T-cell function in response to BLV antigen, PBMC from BLV^+^ cattle were cultured with BLV-gp51 peptide mixture in the presence or absence of anti-PD-1 mAb (5D2), and IFN-γ production and proliferation of lymphocytes were measured. The IFN-γ production in PBMC was upregulated by gp51 peptide mixture relative to Flag peptide (Figure 5a and Additional file 2), indicating that the anti-gp51 immune reaction was induced by gp51 peptide mixture. As expected, PD-1 blockade additionally enhanced IFN-γ production in the presence of a gp51 peptide mix, as compared to the treatment with rat IgG (Figure 5b). The increasing rate of IFN-γ production in PD-1 blockade was correlated with frequencies of PD-1 expression in CD4^+^ T cells (Figure 5c), but not CD8^+^ T cells (data not shown). On the contrary, one of the immunoinhibitory cytokines, IL-10 production was not altered by PD-1 blockade (Figure 5d). Next, proliferation of lymphocytes was analyzed by CFSE staining. Detection of proliferating T cells was difficult because there were very few T cells in cultivated PBMC from BLV-infected cattle causing abnormal B cell proliferation. So, the proliferation of lymphocytes other than B cells was investigated using the gating strategies described in Figure 1a and 2c. PD-1 blockade in PBMC resulted in an increase in the frequencies of CFSE^lo^ IgM^-^ lymphocytes relative to the treatment of control antibody (Figure 5e).

### PD-1 blockade inhibits BLV-gp51 expression and B cell activation

**Figure 6:**
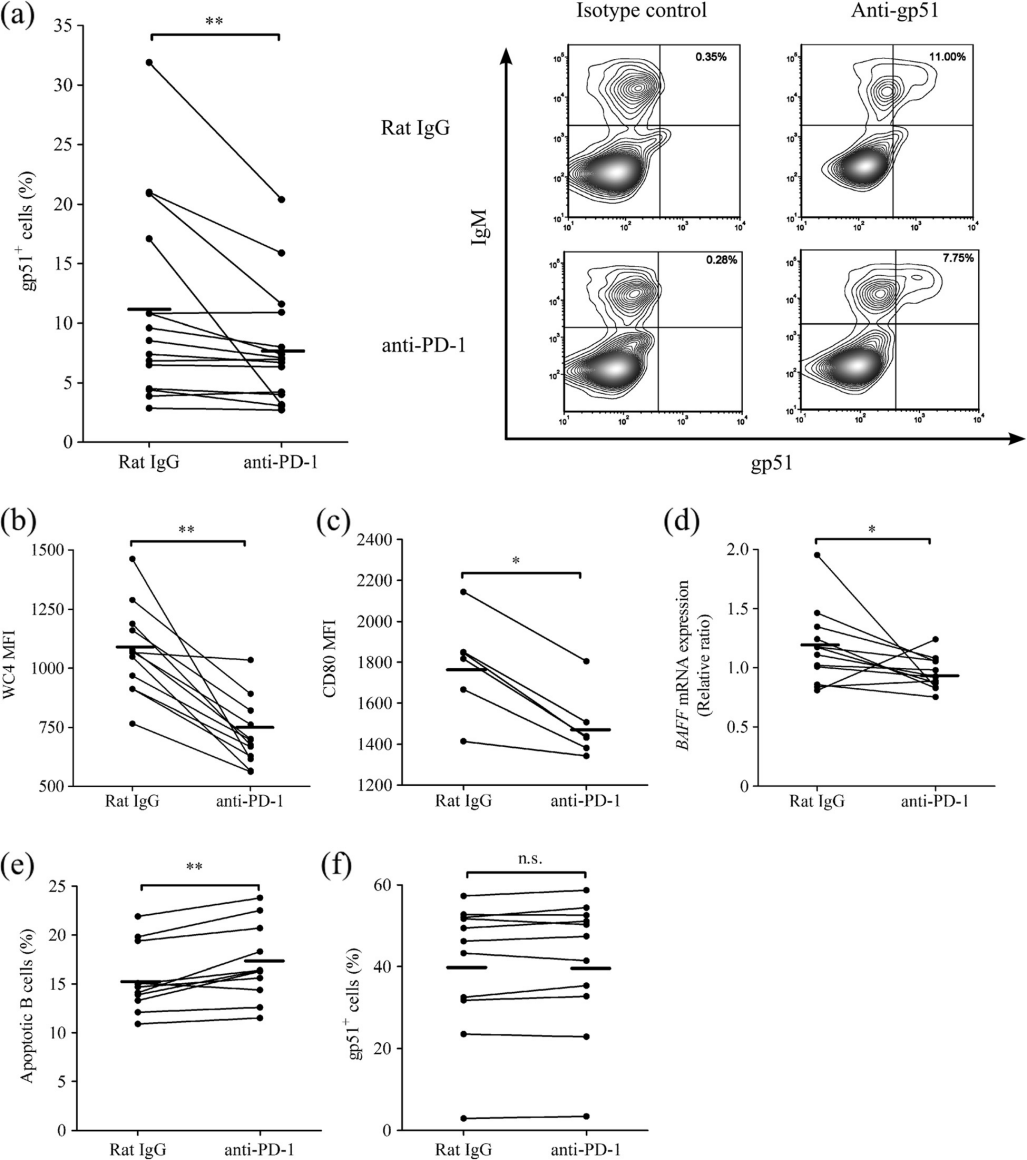
**Effect of PD-1 blockade on gp51 expression and B-cell activation. (a, b, c)** Percentages of gp51-expressing cells (**a**; *n* = 15) and mean fluorescence index (MFI) of WC4 (CD19 like molecule) (**b**; *n* = 12) and CD80 (**c**; *n* = 6) in IgM^+^ B cells were evaluated by flow cytometry in PBMC treated with rat IgG control or anti-PD-1 mAb (20 μg/mL). Representative contour plots showing gp51 expression (right panels) in PBMC treated with rat IgG control (upper panels) or anti-PD-1 mAb (lower panels) is shown in **(a)**. No staining was observed in PBMC stained with isotype control for anti-gp51 mAb (left panels). **(d)** Expression of *BAFF* mRNA was evaluated by real-time PCR (*n* = 12). The results are indicated as relative change to control (no antibody treatment) when the amount of *BAFF* mRNA expression is divided by *GAPDH* mRNA expression. **(e)** Percentages of apoptotic cells in IgM^+^ B cells were measured by flow cytometry. Apoptotic B cells were identified as annexin-V^+^ 7-AAD^-^ cells (*n* = 11). **(f)** Percentages of gp51-expressing cells were evaluated by flow cytometry in isolated B cells cultivated with rat IgG control or anti-PD-1 mAb (*n* = 11). Statistical comparisons between rat IgG control and anti-PD-1 mAb were made using the Wilcoxon matched-pairs test. Differences were considered statistically significant at *P* < 0.05 (*P < 0.05; ** *P* < 0.01).

Next, we evaluated whether PD-1 blockade altered B-cell activation and BLV expression. The incubation of PBMC with gp51 peptide mix in the presence of anti-PD-1 mAb resulted in the reduction of the frequencies of gp51^+^ cells in IgM^+^ B cells (Figure 6a). Because previous reports showed that B cell activation augments viral expression ex vivo [31], we hypothesized that one of the mechanisms responsible for the inhibition of gp51 expression resulting from PD-1 blockade was downregulation of B cell activation. To test this hypothesis, the expression of activation markers, WC4 (CD19 like molecule) and CD80, of B cells were measured. PD-1 blockade resulted in the attenuation of WC4 and CD80 expression in B cells (Figure 6b, c), and, moreover, reduction in the expression of *BAFF* mRNA (Figure 6d), which is an important cytokine for B cell survival [32]. Moreover, the frequencies of apoptotic B cells were also increased in PBMC treated with anti-PD-1 mAb, as compared with control antibody (Figure 6e). Finally, to assess whether PD-1 blockade has a direct effect on B cells, isolated B cells were cultivated with gp51 peptide mix in the presence of anti-PD-1 mAb. As expected, the significant changes in gp51 expression in B cells by the PD-1 blockade were not observed (Figure 6f), suggesting that anti-PD-1 mAb did not directly affect gp51 expression in B cells.

## Discussion

PD-1 on lymphocytes is thought to be a major immunoinhibitory receptor involved in the maintenance of peripheral immune tolerance and immune evasion of tumors and infectious agents [2]. The expression of *PD-1* mRNA was previously analyzed in some animal species by real-time PCR because of lack of specific antibodies [17,20]. However, although the real-time PCR method can be used for quantitative analysis, the method targeting PD-1 requires attention because several splice variants and homologs of the human and mouse *PD-1* gene have been reported [33-35]. *PD-1* mRNA transcripts, in which the third exon of the *PD-1* gene encoding the transmembrane domain was spliced out, encoded a soluble form of PD-1 (sPD-1) [33], and sPD-1 blocked the immunoinhibitory effect of PD-1 expressed on the T cell membrane [34]. Thus, the primer set must be designed carefully to amplify only the membrane-bound form but not the soluble form or other splice variants of *PD-1* mRNA. Expression analysis of membrane-bound PD-1 antigen by flow cytometry is essential to investigate the exact immunoinhibitory effect of PD-1 in immune cells. In this report, we describe the establishment of anti-bovine PD-1 mAb and confirmed the increased frequency of PD-1^+^ T cells in BCBL. We also confirmed the upregulation of IFN-γ production and proliferation and the inhibition of BLV-gp51 expression and B-cell activation in PBMC from BLV^+^ cattle by the treatment with anti-PD-1 mAb.

The molecular weight of both human and bovine PD-1 was expected to be approximately 30 kDa by calculation from their amino acid sequences. However, the band of human PD-1 was found at about 55 kDa in western blotting [26]. Also, in this study, bovine PD-1 was detected at about 68 kDa. From these observations, human PD-1 and bovine PD-1 are thought to be heavily glycosylated, which is consistent with the potential *N*-glycosylation sites of human and bovine PD-1.

CD4^+^ and CD8^+^ T cells are the main targets of study when investigating immunoinhibition induced by PD-1 in human and mouse models. In cattle, CD4^+^ T cells were the main cells that express PD-1 in freshly isolated PBMC, but the expression levels were not so high. In humans and mice, PD-1 expression levels in total CD4^+^ and CD8^+^ T cells are also very low, even when detected by sensitive instruments, such as flow cytometry [26,36]. T cells are divided into naive and different memory subsets by the expression of CD45RO, CD45RA and CCR7, and the memory T cells express high levels of PD-1 [7]. In this study, PD-1 expression in total T cells or B cells was investigated, but detailed expression analysis is needed using many cell markers for clarification whether PD-1^+^ CD4^+^ T cells are the memory T cells or other phenotype.

Stimulation by PMA/ionomycin strongly enhanced PD-1 expression in CD4^+^ and CD8^+^ T cells and B cells. These findings agree with previous reports, which indicated that activated T and B cells express PD-1 antigen [26], perhaps, for the sake of preventing activation-induced cell death. CD25 is the part of IL-2 receptor and identified as the classical activation marker. We hypothesized that PD-1 upregulation occurred in stimulated lymphocytes which was detected by CD25 expression. However, the data revealed that CD4^+^ CD25^-^ T cells also expressed PD-1 and not all CD8^+^ CD25^+^ T cells and IgM^+^ CD25^+^ B cells expressed it 48 h after the stimulation, suggesting that the cell activation detected by CD25 was not the single cause of PD-1 upregulation. More work is needed to clarify the kinetics of induction of PD-1 expression in each cell population.

In chronic infection or tumors, production of inflammatory cytokines and proliferation in response to antigens by pathogen-specific CD4^+^ or CD8^+^ T cells were impaired by the PD-1/PD-L1 pathway. These T cells failed to eradicate the infected cells or cancer cells, and PD-1 was the most appropriate marker of these “exhausted” T cells [9,21]. In this report, BLV infection was chosen as a typical chronic infection and tumor in cattle for PD-1 expression and functional analysis. BLV and HTLV-1 are related deltaretroviruses, and higher percentages of PD-1 expression were observed in CD4^+^ T cells from blood of HTLV-1-infected patients with adult T-cell leukemia [36]. The frequencies of PD-1^+^ cells were also higher in CD4^+^ T cells in blood and both CD4^+^ and CD8^+^ T cells in tumor-bearing LN from BCBL than mesenteric LN from BLV^+^ and BVL^-^ cattle. These data suggest that anergic T cells, which may be specific for lymphoma cells, were increased in total CD4^+^ or CD8^+^ T cells in BCBL. Moreover, the frequency of PD-1^+^ T cells was higher in tumor-bearing LN than peripheral blood in cattle with lymphoma, and the same tendency was reported in patients with metastatic melanoma [37]. Immune tolerance by PD-1 expression in T cells follows T-cell activation by continued epitope recognition in peripheral lymphoid tissue [38-42]. Thus, one can speculate that most tumor infiltrated PD-1^+^ T cells could be specific for lymphoma cells, and present tumor-associated antigen, but fail to be activated properly by the PD-1/PD-L1 pathway, resulting in the immune evasion of BLV-induced lymphoma cells.

To verify the relationship between PD-1 expression in T cells and antigen-specific immunosuppression, PD-1 expression analysis in antigen-specific T cells is required. Indeed, PD-1 upregulation was mainly observed in CD8^+^ T cells specific for pathogens causing chronic infection in various infection and tumors, such as HIV and HTLV infection and mesenteric melanoma [10,37,43]. In this study, PD-1 upregulation in total CD4^+^ and CD8^+^ T cells from BLV^+^ cattle without lymphoma was not observed and PD-1 expression in BLV-specific T cells was not examined. Detection of BLV-specific T cells would enable more detailed analysis of PD-1, although the divergence of gene background of cattle complicates the establishment of MHC-tetramer which is an essential tool for detection. BLV^+^ B cells are not eradicated by the immune system and proliferate in lymphoid tissue in vivo [44], so that BLV-specific T cells from BLV^+^ cattle without lymphoma could recognize antigen continuously, express high PD-1 and lapse into an anergic state by PD-L1 expression in BLV^+^ B cells.

In BLV^+^ cattle with advanced stage of the disease, proliferation of CD4^+^ T cells in response to BLV proteins, such as gag and env, is impaired [6]. In this study, PD-1 blockade enhanced the IFN-γ production in PBMC in response to the gp51 peptide mix. Moreover, the increasing rate of IFN-γ production was correlated with percentages of PD-1^+^ cells in CD4^+^ T cells, suggesting that PD-1 blockade invigorated the function of PD-1^+^ CD4^+^ T cells, perhaps gp51-specific T cells. Meanwhile, IL-10 production was not altered in PBMC by treatment with anti-PD-1 mAb. Blockade of PD-1/PD-L1 pathway does not seem to upregulate all functions of PD-1^+^ T cells.

On the contrary, PD-1 blockade resulted in the inhibition of gp51 expression, reduced the expression of activation marker of B cells, WC4 and CD80 and increased B-cell apoptosis. Although the mechanism of BLV-gp51 expression both in vitro cultivation and in vivo is not dissolved, B-cell activation by immune-mediated stimulation is known as the important factor for activation of viral protein synthesis [31]. This study did not demonstrate that T cells reactivated by anti-PD-1 mAb have a direct effect on the B-cell activity. However, anti-PD-1 mAb did not directly affect B cells, because recognizable PD-1 expression was not observed in B cells and PD-1 treatment of isolated B cells did not alter gp51 expression. Changes in the cytokine environment, such as reduction of BAFF or activation of cytotoxic T cells induced by PD-1 blockade could create a disadvantageous environment for B cells, subsequently resulting in the reduction of gp51, WC4 and CD80 expression and increased B-cell apoptosis.

PD-1 is expected to be a potential target for reinvigorating the function of exhausted T cells. Many researchers have investigated antibody treatment that blocks the PD-1/PD-L1 pathway [11,43,45], and clinical trials in patients with cancer who were administered anti-PD-1 antibody are now ongoing [13,14]. Anti-bovine PD-1 mAb upregulated IFN-γ production in PBMC from BLV^-^ cattle, indicating that the immune reactivation by PD-1 blockade is not a limited phenomenon in BLV infection. Although it is unknown whether PD-1^+^ T cells in BLV^-^ cattle are specific for some pathogens, there is the possibility that treatment with these anti-PD-1 mAb could be applied to new targets of therapy for many types of infection in cattle via upregulation of immune responses. Moreover, the mAb could promote research regarding bovine immunology and clarify the mechanisms of diseases that induce immunosuppression.

## Abbreviations

BCBL: Bovine leukemia virus-infected cattle with B-cell lymphoma; BLV: Bovine leukemia virus; BLV+: Bovine leukemia virus-infected; BLV-: Bovine leukemia virus-uninfected; CHO: Chinese hamster ovary; ELISA: Enzyme-linked immunosorbent assay; HRP: Horse radish peroxidase; IFN-γ: Interferon-gamma; IL: Interleukin; LN: Lymph node; mAb: Monoclonal antibody; pAb: Polyclonal antibody; PBMC: Peripheral blood mononuclear cells; PBS: Phosphate-buffered saline; PBS-T: 0.05% Tween 20 in phosphate-buffered saline; PD-1: Programmed death-1; PD-1-Ig: Soluble PD-1-bovine IgG1 fusion protein; PD-L1: Programmed death-ligand 1; PMA: Phorbol 12-myristate acetate; PWM: Pokeweed mitogen; SDS-PAGE: Sodium dodecyl sulfate-polyacrylamide gel electrophoresis; sPD-1: Soluble form of programmed death-1.

## Competing interests

The authors declare that they have no competing interests.

## Authors’ Contributions

RI performed all of the studies contained in this manuscript, analyzed data, and drafted the manuscript. SK participated in the experimental design, analyzed data, and helped to draft the manuscript. TO participated in some experiments and sample collection. KY, CN, and YS participated in the experiments involving expression and purification of soluble proteins, and reviewed the manuscript. SM helped with the experimental design and data interpretation. KO supervised the study and reviewed the manuscript. All authors read and approved the final manuscript.

## Supplementary Material

Additional file 1 **An example of BLV-gp51 expression in freshly isolated and cultivated lymphocytes.** Values in the quadrant indicate the percentage of gp51^+^ cells in lymphocytes. Click here for file

Additional file 2 **IFN-γ production in PBMC cultivated with peptide mixture and anti-PD-1 mAb treatment.** Error bars represent the SEM of the means among the seven cattle. Statistical comparisons were made using one-way ANOVA with the Tukey’s test. Click here for file
