# Supplementary figures and images for: Blockade of bovine PD-1 increases T cell function and inhibits bovine leukemia virus expression in B cells in vitro
Source: Vet Res. 2013 Jul 22;44(1):59. doi: 10.1186/1297-9716-44-59 (PMC3726328; doi:10.1186/1297-9716-44-59)

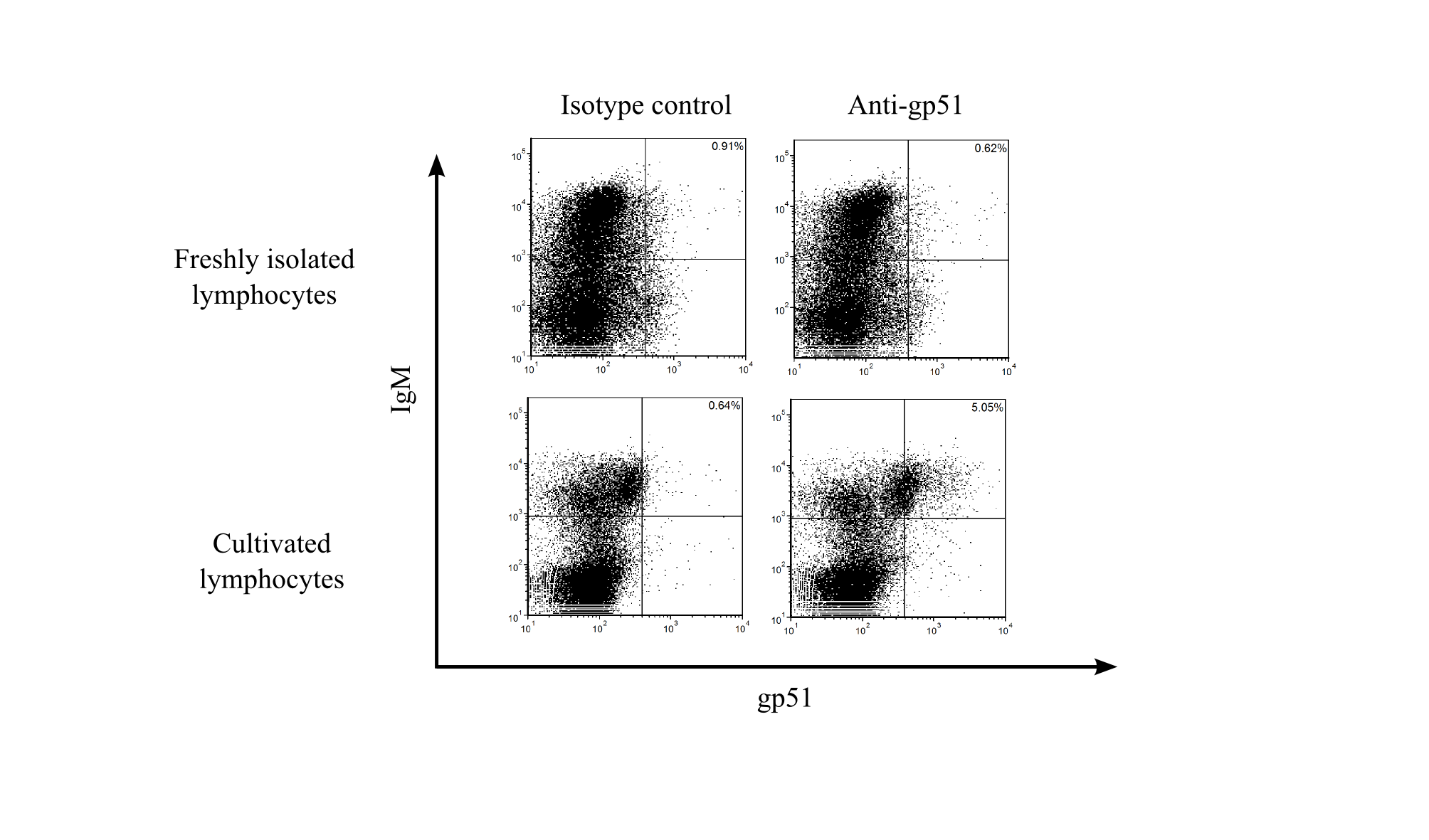

Supplement: Additional file 1 — An example of BLV-gp51 expression in freshly isolated and cultivated lymphocytes. Values in the quadrant indicate the percentage of gp51+ cells in lymphocytes. [file 1297-9716-44-59-S1.png]

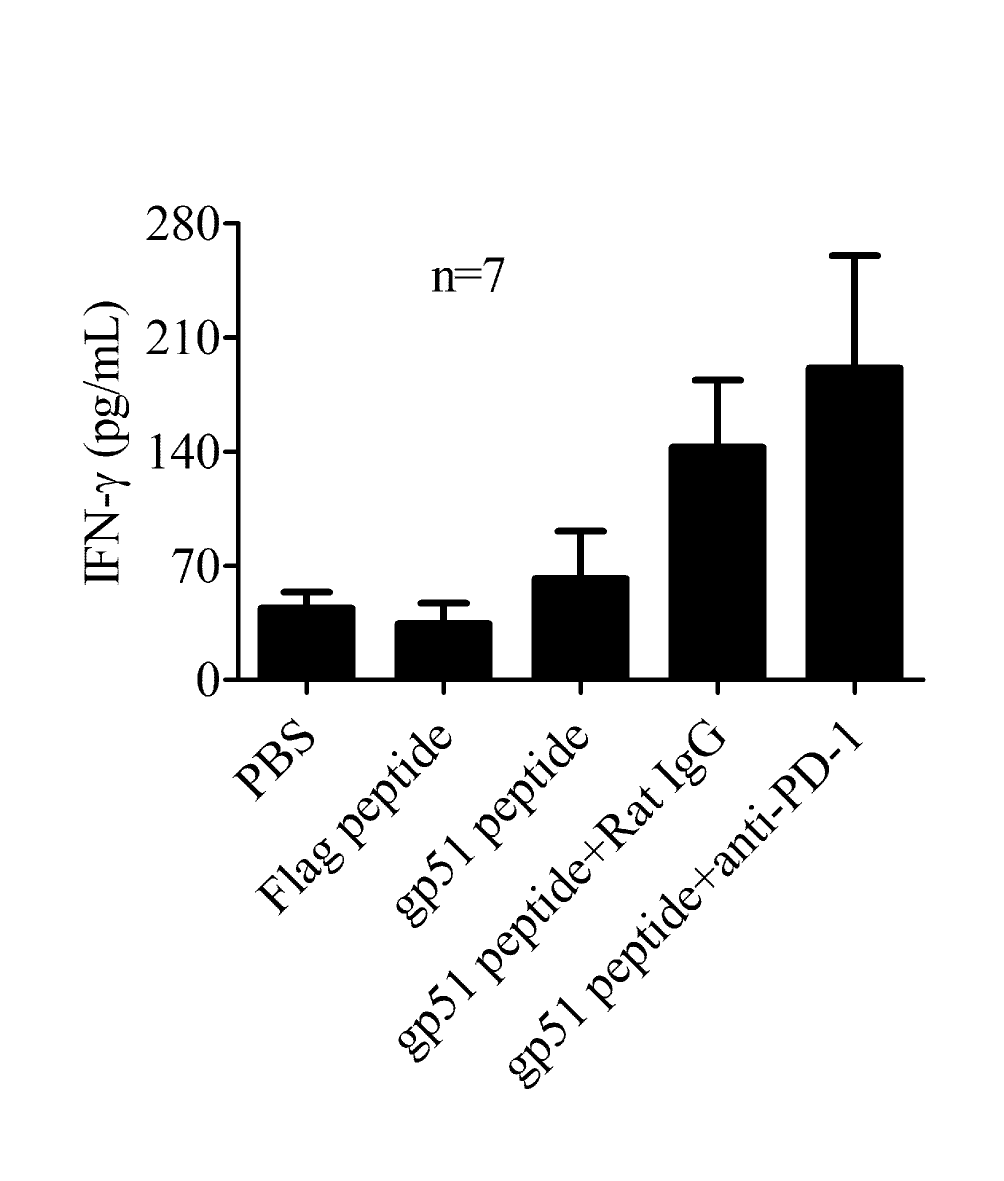

Supplement: Additional file 2 — IFN-γ production in PBMC cultivated with peptide mixture and anti-PD-1 mAb treatment. Error bars represent the SEM of the means among the seven cattle. Statistical comparisons were made using one-way ANOVA with the Tukey’s test. [file 1297-9716-44-59-S2.png]
